# Investigating Aberrant Salience in Autism Spectrum Disorder and Psychosis Risk: A Cross‐Group Analysis

**DOI:** 10.1111/eip.70099

**Published:** 2025-10-06

**Authors:** Federico Fiori Nastro, Martina Pelle, Alice Clemente, Fernando Corinto, Davide Prosperi Porta, Yael Sonnino, Carmine Gelormini, Giorgio Di Lorenzo, Michele Ribolsi

**Affiliations:** ^1^ Chair of Psychiatry, Department of Systems Medicine Tor Vergata University of Rome Rome Italy; ^2^ IRCCS Fondazione Santa Lucia Rome Italy; ^3^ Unit of Neurology, Neurophysiology, Neurobiology and Psychiatry, Department of Medicine Campus Bio‐Medico University Rome Italy; ^4^ Institute of Biomedical and Neural Engineering Reykjavik University Reykjavik Iceland; ^5^ Department of Life Science, Health, and Health Professions Link Campus University Rome Italy

**Keywords:** autism spectrum disorder, comorbidity, neurodevelopmental disorders, psychosisattenuated psychosis syndrome

## Abstract

**Aim:**

This study investigates the expression of aberrant salience (AS) in individuals with autism spectrum disorder (ASD), those at clinical high risk for psychosis (CHR‐P) and help‐seeking individuals without formal diagnoses.

**Methods:**

Ninety‐nine participants, 44 males and 55 females (age range 17–39 years), met the inclusion criteria of absence of major neurological disorders, intellectual disabilities or substance‐related conditions. None were receiving antipsychotic treatment. Based on clinical evaluations, participants were categorised into three groups: ASD (*n* = 23), Attenuated Psychosis Syndrome (APS) (*n* = 27) and help‐seekers (*n* = 49). The Aberrant Salience Inventory (ASI) was administered.

**Results:**

Significant differences were observed in ASI total and subscale scores among groups. Post hoc analyses showed significantly higher ASI scores in the ASD and APS groups compared to help‐seekers, but no significant differences between ASD and APS.

**Conclusions:**

These findings suggest an overlap in AS processing between ASD and psychosis‐spectrum conditions, supporting AS as a transdiagnostic construct.

## 1 Introduction

Autism Spectrum Disorder (ASD) is a lifelong neurodevelopmental condition characterised by two key symptoms: persistent deficits in social communication/interaction and restricted, repetitive patterns of behaviour and abnormal sensory response (American Psychiatric Association 2022). Recent literature reports a global rise in the prevalence and incidence of ASD, with a potential 75‐fold rise from 1988–1989 to 2010–2015 (Watkins and Angus‐Leppan 2022). Diagnosing ASD remains challenging due to its heterogeneous clinical presentations, requiring both early recognition by caregivers and confirmation through multidisciplinary assessment (Hyman et al. 2020). Additionally, most individuals with ASD present comorbid psychiatric conditions, increasing their risk for severe mental illness and further impairing long‐term outcomes (Hossain et al. 2020; Pelle et al. 2025).

Although Schizophrenia spectrum disorder (SSD) and ASD are currently regarded as distinct conditions, increasing evidence highlights significant overlaps between the two spectra. Commonalities in genetic studies, neuroimaging data, clinical signs, cognitive features and social skills impairments have been identified (Vaquerizo‐Serrano et al. 2022; Ribolsi, Fiori Nastro, et al. 2022; Riccioni et al. 2022; Solomon et al. 2011). Literature reports that up to 34.8% of individuals with ASD may exhibit psychotic symptoms, while autistic traits have been observed in 3.6% to 60% of SSD patients (Chisholm et al. 2015). This overlap often results in ASD patients meeting Clinical‐high risk for psychosis (CHR‐P) criteria, complicating the distinction between autistic traits and attenuated psychotic symptoms, particularly in adolescents (Ribolsi, Fiori Nastro, et al. 2022; Fiori Nastro, Esposto, et al. 2023).

In the early 2000s, Kapur proposed that psychotic symptoms may result from altered salience processing (Kapur 2003). Aberrant salience (AS) refers to the neurocognitive tendency to assign inappropriate significance to otherwise irrelevant stimuli within perceptual, cognitive or emotional contexts. While the construct of salience encompasses the capacity of external or internal stimuli to capture an individual's attention or acquire motivational relevance, the term aberrant denotes a pathological deviation from normative salience assignment. In the last few years, there has been a renewed interest in salience alterations and their relevance to psychosis psychopathology (Kapur 2003; Azzali et al. 2022; Fiori Nastro, Pelle, et al. 2023; Merola et al. 2025).

AS is posited to constitute a fundamental mechanism underpinning the formation of psychotic symptoms, most notably delusions and hallucinations, through the maladaptive integration of anomalously salient experiences into personal meaning structures.

Conceptualization of AS has identified multiple sub‐constructs that reflect distinct but interrelated components of this phenomenon, typically measured using the Aberrant Salience Inventory (ASI) (see Section 2). A five‐structure model of the ASI has been proposed (Cicero et al. 2010; LeIli et al. 2015): ‘Feelings of increased significance’ reflects a heightened sense of meaningfulness or importance attached to innocuous stimuli; ‘Sharpening of senses’ encompasses reports of enhanced sensory acuity, often described as heightened perception of sounds; ‘*Impending understanding*’ captures the experience of feeling on the verge of a profound insight or revelation; ‘*Heightened emotionality*’ reflects elevated anxiety and emotional arousal in response to the perceived increased significance of stimuli during the prodromal phase of psychosis; ‘*Heightened cognition*’ refers to the subjective experience of unusually intense thought processes, often accompanied by a sense of involvement in meaningful or hidden events.

The study of AS is enhancing the knowledge and the clinical understanding of the psychosis risk continuum, spanning from CHR‐P states to full‐blown psychosis. This makes AS a crucial psychopathological marker for identifying those at increased risk of developing psychosis, particularly youth seeking help (Fiori Nastro, Pelle, et al. 2023; Fiori Nastro, Clemente, et al. 2024; Lisi et al. 2021).

Recent studies have examined salience alterations in relation to autism symptomatology, focusing on the salience network (SN) and its alteration in connectivity with other brain regions using resting‐state fMRI (Jao Keehn et al. 2021; Green et al. 2016; Attanasio et al. 2024). However, to the best of our knowledge, no studies to date have investigated Kapur's construction of AS in relation to autism symptomatology through the administration of the ASI.

To enhance understanding of the psychopathological overlap between ASD and SSD and identify clinical and prognostic markers that differentiate the two spectra, this exploratory study aims to examine the prevalence of AS in individuals with ASD, in comparison to those at CHR‐P and help‐seeking individuals.

## 2 Materials and Methods

We conducted a cross‐sectional study of 99 subjects enrolled from the psychiatry outpatient units of the University Hospital Campus Bio‐Medico and the Policlinico Tor Vergata in Rome.

The adopted exclusion criteria were: age over 40 years or under 16 years; intelligence quotient equal to or less than 70, established by the Wechsler Adult Intelligence Scale‐Revised (WAIS‐R) (Franzen 2000); concurrent presence of relevant neurological comorbidities (e.g., epilepsy, concussion, or traumatic brain injury); current substance use disorder; past or undergoing use of antipsychotic treatment.

### 2.1 Participants

Twenty‐three subjects had a diagnosis of ASD (10 females, 43.48%; age range 17–39, mean ± SD, 27.04 ± 5.91). All participants were assessed using the Autism Diagnostic Observation Schedule, Second Edition (ADOS‐2) (Lord et al. 2012). The ADOS‐2 was administered by expert clinicians familiar with its use.

Twenty‐seven CHR‐P (14 females, 51.85%; age range 17–30, mean ± SD, 22.19 ± 3.59) were enrolled. CHR‐P was defined using the criteria for Attenuated Psychosis Syndrome (APS), as introduced in the Diagnostic and Statistical Manual of Mental Disorders‐Fifth Edition (DSM‐5)– Research Appendix Section III (American Psychiatric Association 2022; Salazar de Pablo et al. 2020). The Structured Interview for Prodromal Syndromes (SIPS) and the Scale of Prodromal Syndromes (SOPS) (Miller et al. 2003) were also administered to confirm the APS condition. APS subjects were longitudinally followed at Policlinico Tor Vergata (Fiori Nastro, Clemente, et al. 2024). All 27 CHR‐P individuals were followed up after 12 months and classified as Converters or Non‐Converters based on whether they transitioned to full‐blown psychosis. Eight participants converted to psychosis (2 females, 25%; age range: 18–25 years; mean ± SD: 21.4 ± 2.56), while 19 did not convert (12 females, 63.15%; age range: 17–30 years; mean ± SD: 22.5 ± 3.95).

Finally, 49 help‐seekers (31 females, 63.26%; age range 19–29, mean ± SD, 23.08 ± 2.48) were enrolled. Help‐seekers accessed the psychological counselling service for university students (Fiori Nastro, Pelle, et al. 2024) and did not meet criteria for any psychiatric or neurodevelopmental disorders, or APS condition. All help‐seekers included in this study scored ≤ 2 on the Prodromal Questionnaire (PQ‐16) (Parabiaghi et al. 2024) and ≤ 6 on the Beck Depression Inventory‐II (BDI‐II) (Beck et al. 1996), indicating minimal symptomatology. Clinical evaluation and the Mini International Neuropsychiatric Interview (MINI) (Hyun et al. 2007) confirmed the absence of categorical mental disorders in this group.

### 2.2 Questionnaires

The ASI (Cicero et al. 2010; LeIli et al. 2015) was administered to measure AS. ASI is organised into five subscales: ‘*Feelings of increased significance*’, ‘*Sharpening of senses*’, ‘*Impending understanding*’, ‘*Heightened emotionality*’ and ‘*Heightened cognition*’ (LeIli et al. 2015). The ASI showed excellent internal consistency (Cronbach's *α* = 0.89) and strong convergent validity. The ASI Italian version (LeIli et al. 2015) replicated the scale's factorial structure and demonstrated high internal consistency (Cronbach's *α* = 0.89) and good test–retest reliability over 15 days (*r* = 0.96, *p* < 0.001). The scale effectively discriminated between psychiatric patients and controls, as well as between patients with and without psychotic symptoms, confirming its clinical utility and robustness across languages and populations.

Research has consistently shown higher ASI scores in psychotic and CHR‐P compared to healthy controls (Fiori Nastro, Pelle, et al. 2023; Fiori Nastro, Clemente, et al. 2024; Merola et al. 2023). To our knowledge, this is the first study to use ASI in an ASD population. Given the correlation and overlap between ASD and psychosis, we would have expected individuals with ASD to exhibit ASI scores intermediate between those of APS and help‐seekers.

### 2.3 Statistical Analysis

Statistical analysis was performed using R (R Core Team R 2021). Group differences were evaluated using non‐parametric methods. A Kruskal–Wallis test was conducted via the kruskal_test() function from the ‘coin’ package, employing an approximate distribution with 10 000 resamples to derive *p*‐values. For post hoc analysis, the pSDCFlig() function from the ‘NSM3’ package was applied, implementing the Dwass–Steel–Critchlow–Fligner (DSCF) procedure with 10 000 resampling iterations to derive robust *p*‐values. The significance level was set at 0.05.

Effect sizes for DSCF pairwise post hoc comparisons were calculated using Pearson's *r* (*r* = *Z*/√(*n*_1_ + *n*_2_)), where *n*_1_ and *n*_2_ represent the sample sizes of the two groups compared. *R* values of 0.10–0.30 were considered small, 0.30–0.50 moderate and ≥ 0.50 large.

Moreover, to control for potential confounding continuous variables, we conducted an additional non‐parametric ANCOVA including age and years of education as covariates. Non‐parametric ANCOVA was conducted via the aov() function from the ‘sm’ package.

Figures were realised using JASP, version 0.18.1, for macOS (JASP Team 2025).

## 3 Results

### 3.1 Participants

Ninety‐nine subjects (55 females, 55.55%; age range, 17–39 years; mean ± SD, 23.75 ± 4.2) were enrolled.

Participants had an average education level of 14.62 years (SD ±2.75). Sixteen participants (16.16%) reported cannabis use and 53 (53.53%) resided in urban areas. Additionally, 13 individuals (13.13%) (six APS and seven ASD) were undergoing pharmacological treatment with antidepressants, mood stabilisers or benzodiazepines at the time of the first evaluation.

Detailed descriptive and univariate statistics for the sociodemographic characteristics of the three groups are provided in Table 1.

**TABLE 1:** Descriptive and univariate statistics of sociodemographic characteristics in the ASD vs. APS vs. Help‐seekers groups.

	ASD (*n* = 23; 23.23%)	APS (*n* = 27; 27.27%)	Help‐seekers (*n* = 49; 49.5%)	Statistics	
				Value^a^	*p* ^b^
Age	27.04 ± 5.91	22.19 ± 3.59	23.08 ± 2.48	2	**0.002**
Education	13.22 ± 2.56	13.22 ± 3.23	16.06 ± 1.65	2	< **0.001**
Gender					
F	10 (43.48%)	14 (51.85%)	31 (63.26%)	2.69	0.252
M	13 (56.52%)	13 (48.15%)	18 (36.74%)		
Cannabis use					
Yes	1 (4.35%)	8 (29.63%)	7 (14.29%)	6.11	0.052
No	22 (95.65%)	19 (70.37%)	42 (85.71%)		
Urban area					
Yes	14 (60.87%)	14 (51.85%)	25 (51.02%)	0.65	0.726
No	9 (39.13%)	13 (48.15%)	24 (48.98%)		
Pharmacological therapy					
Yes	7 (30.44%)	6 (22.22%)	0	15.4	< **0.001**
No	16 (69.56%)	21 (77.78%)	49 (100%)		

*Note:* Significant *p*‐values are in bold.

Abbreviations: F, female; M, male.

^a^ Continuous variables were analysed using the Kruskal–Wallis test, while categorical variables were compared using a contingency table and *χ* ^2^.

^b^ The Chi‐square *p*‐value has been adjusted using Fisher's exact test. Age and education are presented in mean years with standard deviation.

### 3.2 Group Differences

The three groups did not differ in gender, cannabis use, or urban residency but showed significant differences in age and years of education. The ASD group was the oldest, with a mean age of 27.04 years (SD = 5.91), compared to the APS group (22.19 ± 3.59) and help‐seekers (23.08 ± 2.48). Conversely, help‐seekers had slightly more years of education (16.06 ± 1.65), nearly 3 years more than both the ASD (13.22 ± 2.56) and APS (13.22 ± 3.23) groups. Moreover, statistically significant differences in pharmacological treatment were observed among the three groups. All treatments were prescribed by general practitioners prior to enrollment. As stated in the exclusion criteria, none of the individuals in the ASD or APS groups had a history of antipsychotic treatment or were receiving antipsychotics at the time of assessment. Moreover, none of the participants met the criteria for a diagnosis of a major depressive episode or other mood disorders. The help‐seekers were entirely untreated.

Applying the Kruskal‐Wallis test, differences in ASI total and three out of five ASI subscale scores *(*‘*Sense Sharpening*’, ‘*Impending Understanding*’ and ‘*Heightened Cognition*’) were observed among the three groups. The ASI subscales ‘Feelings of Increased Significance’ and ‘*Heightened Emotionality*’ did not reach statistical significance. Descriptive and univariate statistics for the ASI are presented in Table 2a.

**TABLE 2a:** Descriptive and univariate statistics for the ASI scores in the ASD vs. APS vs. help seeker groups.

	ASD (*n* = 23; 23.23%)	APS (*n* = 27; 27.27%)	Help‐seekers (*n* = 49; 49.5%)	Statistics^a^	
				H	*p*
ASI total score	14.04 ± 7.71	12.22 ± 10.01	7.65 ± 5.03	9.308	**0.008**
Feeling of increased significance	4.17 ± 2.22	3.59 ± 2.66	3.38 ± 2.07	2.295	0.322
Sense sharpening	2.09 ± 1.56	1.70 ± 1.77	0.45 ± 0.68	25.26	**< 0.001**
Impending understanding	2.65 ± 1.82	2.41 ± 1.82	1.32 ± 1.30	10.994	**0.003**
Heightened emotionality	2.96 ± 2.14	2.59 ± 2.24	1.69 ± 1.29	5.622	0.056
Heightened cognition	2.21 ± 1.65	1.92 ± 2.27	0.82 ± 0.99	12.403	**0.001**

*Note:* Significant *p*‐values are in bold.

^a^ Kruskall–Wallis test. ASI scores are presented as means with standard deviations.

The non‐parametric ANCOVA, controlling for age and education as covariates, yielded results comparable to those obtained in the primary Kruskal–Wallis tests, suggesting that our findings are robust to these demographic differences. Non‐parametric ANCOVA results are summarised in the Table S1.

**FIGURE 1:**
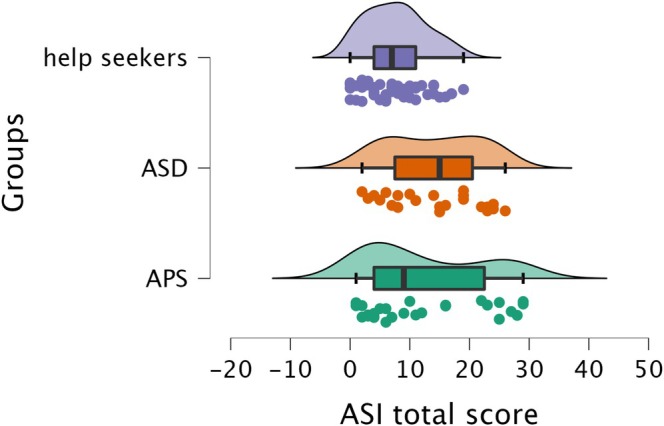
The raincloud plots depict mean differences in ASI total scores among autism spectrum disorder (ASD), attenuated psychosis syndrome (APS) and help‐seekers.

Post hoc analysis using the Dwass–Steel–Critchlow–Fligner test revealed that ASI total and subscale scores did not significantly differ between the ASD and APS groups. On the other hand, ASD scored significantly higher than help‐seekers on the ASI total and three ASI subscale scores (‘*Sense Sharpening*’, ‘*Impending Understanding*’ and ‘*Heightened Cognition*’). The APS had significantly higher scores than help‐seekers in only two ASI subscales (‘*Sense Sharpening*’, ‘*Impending Understanding*’). Post hoc analyses are presented in Table 2b–g, while Figure 1 reports differences in ASI total scores among the three groups.

**TABLE 2b–g:** Post hoc pairwise comparisons with Dwass–Steel–Critchlow–Fligner test.

	W	*p*	*r* (effect size)	*p*
*(b) ASI total score*				
APS vs. ASD	1.253	0.650	0.177	0.654
APS vs. help‐seekers	−1.937	0.363	−0.222	0.362
ASD vs. help‐seekers	−4.575	**0.003**	−0.539	**0.002**
*(c) Feeling of increased significance*				
APS vs. ASD	1.352	0.604	0.191	0.605
APS vs. help‐seekers	−0.435	0.949	−0.050	0.952
ASD vs. help‐seekers	−2.201	0.264	−0.260	0.2603
*(d) Sense sharpening*				
APS vs. ASD	1.548	0.516	0.219	0.517
APS vs. help‐seekers	−4.938	**< 0.001**	−0.566	**0.0013**
ASD vs. help‐seekers	−6.664	**< 0.001**	−0.785	0
*(e) Impending understanding*				
APS vs. ASD	0.559	0.919	0.079	0.921
APS vs. help‐seekers	−3.547	**0.032**	−0.407	**0.0301**
ASD vs. help‐seekers	−4.109	**0.008**	−0.484	**0.0085**
*(f) Heightened emotionality*				
APS vs. ASD	0.920	0.799	0.130	0.801
APS vs. help‐seekers	−2.040	0.318	−0.234	0.318
ASD vs. help‐seekers	−3.241	0.054	−0.382	0.058
*(g) Heightened cognition*				
APS vs. ASD	1.604	0.493	0.227	0.492
APS vs. help‐seekers	−2.296	0.234	−0.263	0.227
ASD vs. help‐seekers	−5.229	**< 0.001**	−0.616	**< 0.0001**

*Note:* Significant *p*‐values are in bold.

Individuals meeting APS criteria were longitudinally assessed after 12 months and classified as Converters or Non‐Converters based on their transition to full‐blown psychosis. Using the baseline scores for individuals with APS, the Kruskal–Wallis test revealed statistically significant differences in ASI total and subscales scores across the four groups (ASD vs. Converters vs. Non‐Converters vs. help‐seekers). Post hoc analysis revealed significant differences between Converters and help‐seekers, as well as between individuals with ASD and help‐seekers. Converters scored significantly higher than help‐seekers on the ASI total score and all subscales. The ASD group also showed significantly higher scores than help‐seekers on the ASI total score and three subscales (‘*Sense Sharpening*’, ‘*Impending Understanding*’ and ‘*Heightened Cognition*’). Non‐Converters differed from help‐seekers only on the ‘*Sense Sharpening*’ subscale, with help‐seekers scoring higher. No significant differences were observed among the remaining groups. Results are summarised in Table 3a and Table 3b–g.

**TABLE 3a:** Descriptive and univariate statistics for the baseline ASI scores in the ASD vs. Converters vs. Non‐Converters vs. help seekers groups.

	ASD (*n* = 23; 23.23%)	Converters (*n* = 8; 8.08%)	Non‐Converters (*n* = 19; 19.19%)	Help‐seeker (*n* = 49; 49.5%)	Statistics^a^	
					H	*p*
ASI total score	14.04 ± 7.71	20.00 ± 9.90	8.94 ± 8.27	7.65 ± 5.03	17.699	**< 0.001**
Feeling of increased significance	4.17 ± 2.23	5.50 ± 2.39	2.79 ± 2.39	3.39 ± 2.07	10.486	**0.0119**
Sense sharpening	2.09 ± 1.56	2.75 ± 2.18	1.26 ± 1.41	0.45 ± 0.67	27.176	**< 0.001**
Impending understanding	2.65 ± 1.82	3.75 ± 1.39	1.84 ± 1.71	1.33 ± 1.30	17.323	**< 0.002**
Heightened emotionality	2.96 ± 2.14	4.25 ± 2.25	1.90 ± 1.88	1.69 ± 1.29	12.393	**0.005**
Heightened cognition	2.22 ± 1.65	3.75 ± 2.43	1.16 ± 1.74	0.82 ± 0.99	20.956	**< 0.001**

*Note:* Significant *p*‐values are in bold.

^a^ Kruskall–Wallis test. ASI scores are presented as means with standard deviations.

**TABLE 3b–g:** Post hoc pairwise comparisons with Dwass–Steel–Critchlow–Fligner test.

	W	*p*	*r* (effect size)	*p*
*(b) ASI total score*				
Non‐Converters vs. Converters	3.234	0.093	0.622	0.097
Non‐Converters vs. ASD	3.095	0.119	0.477	0.121
Non‐Converters vs. help‐seekers	0.213	0.999	0.026	0.999
Converters vs. ASD	−2.621	0.248	−0.471	0.246
Converters vs. help‐seekers	−4.458	**0.005**	−0.591	**0.006**
ASD vs. help‐seekers	−4.575	**0.004**	−0.539	**0.004**
*(c) Feeling of increased significance*				
Non‐Converters vs. Converters	3.134	0.115	0.603	0.108
Non‐Converters vs. ASD	2.928	0.158	0.452	0.152
Non‐Converters vs. help‐seekers	1.674	0.637	0.203	0.641
Converters vs. ASD	−2.117	0.436	−0.380	0.432
Converters vs. help‐seekers	−3.735	**0.037**	−0.495	**0.033**
ASD vs. help‐seekers	−2.201	0.398	−0.260	0.397
*(d) Sense sharpening*				
Non‐Converters vs. Converters	2.215	0.393	0.426	0.393
Non‐Converters vs. ASD	2.660	0.230	0.411	0.229
Non‐Converters vs. help‐seekers	−3.773	**0.030**	−0.458	**0.031**
Converters vs. ASD	−1.134	0.853	−0.204	0.860
Converters vs. help‐seekers	−4.391	**0.006**	−0.582	**0.007**
ASD vs. help‐seekers	−6.664	**< 0.001**	−0.785	0
*(e) Impending understanding*				
Non‐Converters vs. Converters	3.473	0.057	0.669	0.057
Non‐Converters vs. ASD	1.854	0.548	0.286	0.554
Non‐Converters vs. help‐seekers	−1.456	0.731	−0.177	0.734
Converters vs. ASD	−2.029	0.471	−0.364	0.474
Converters vs. help‐seekers	−5.080	**< 0.001**	−0.673	**< 0.001**
ASD vs. help‐seekers	−4.109	**0.014**	−0.484	**0.014**
*(f) Heightened emotionality*				
Non‐Converters vs. Converters	3.357	0.073	0.646	0.076
Non‐Converters vs. ASD	2.354	0.335	0.363	0.343
Non‐Converters vs. help‐seekers	−0.059	1.000	−0.007	1
Converters vs. ASD	−2.074	0.455	−0.373	0.455
Converters vs. help‐seekers	−4.219	**0.010**	−0.559	**0.010**
ASD vs. help‐seekers	−3.241	0.092	−0.382	0.093
*(g) Heightened cognition*				
Non‐Converters vs. Converters	3.584	**0.049**	0.690	**0.047**
Non‐Converters vs. ASD	3.458	0.063	0.533	0.06
Non‐Converters vs. help‐seekers	−0.166	0.999	−0.020	1
Converters vs. ASD	−2.396	0.318	−0.431	0.315
Converters vs. help‐seekers	−4.568	**0.004**	−0.605	**0.004**
ASD vs. help‐seekers	−5.229	**< 0.001**	−0.616	**< 0.001**

*Note:* Significant *p*‐values are in bold.

The non‐parametric ANCOVA, controlling for age and education as covariates, yielded similar results. Details are provided in the Table S2.

**FIGURE 2:**
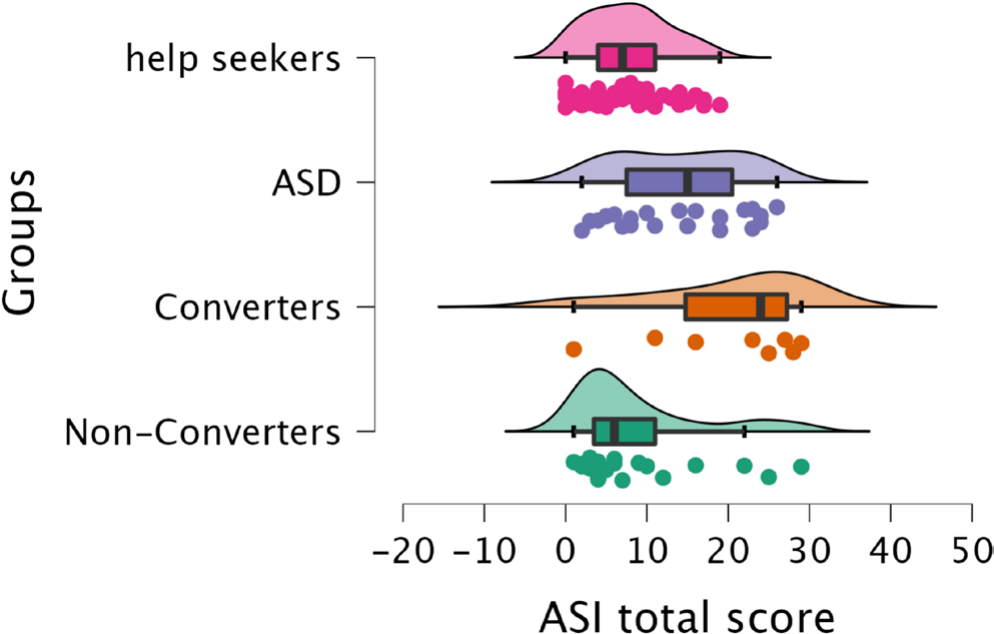
Raincloud plots depict mean differences in ASI total scores among individuals with autism spectrum disorder (ASD), individuals with attenuated psychosis syndrome who converted to full‐blown psychosis at the 12‐month evaluation (Converters), individuals who did not convert (Non‐Converters) and help‐seekers.

Figure 2 illustrates ASI score differences across the four groups.

## 4 Discussion

This is the first study investigating differences in AS expression across individuals with ASD, APS and help‐seekers. Our analysis reveals statistically significant differences in ASI scores in the three groups, with ASD obtaining the highest ASI mean scores. ASI has demonstrated satisfactory psychometric properties, including high test–retest reliability and excellent internal consistency (LeIli et al. 2015; Merola et al. 2023), supporting its use in early identification strategies in clinical populations and in mental health services (Cicero et al. 2010).

The post hoc analysis revealed that ASI scores differentiate individuals with ASD from help‐seekers. Autistic individuals exhibit a higher risk of developing psychosis compared to the general population (Varcin et al. 2022). As previously noted, epidemiological evidence indicates that up to 34.8% of individuals with ASD may exhibit psychotic symptoms, while autistic traits have been reported in 3.6% to 60% of individuals with SSD (Chisholm et al. 2015; Kincaid et al. 2017). This overlap might explain ASD's higher ASI scores compared to help‐seekers, suggesting that autistic individuals could be particularly susceptible to psychotic‐like experiences. The higher ASI scores in the ASD group may reflect underlying neurocognitive mechanisms such as sensory hypersensitivity, heightened interoceptive awareness and atypical processing of salience‐related stimuli, features that have been linked to both ASD and psychosis‐prone phenotypes (Pellicano and Burr 2012; Abu‐Akel et al. 2017).

On the other hand, APS and help‐seekers only differed significantly on two ASI subscale scores: ‘*Sense Sharpening*’ and ‘*Impending Understanding*’. Correlations between ASI subscales and psychotic symptoms have been observed, with positive symptoms and general psychopathology being particularly associated with ‘*Sense Sharpening*’ (Pugliese et al. 2022). Moreover, ‘*Impending Understanding*’ has been conceptualised as a pivotal step in the progression from a pre‐delusional state to fully developed psychotic experiences (Raballo et al. 2019). Since previous research has highlighted comparable levels of AS between CHR‐P and first‐episode psychosis (FEP) (Poletti et al. 2022), a consistent differentiation between APS and help‐seekers would have been expected. The absence of broader differences between these two groups warrants further consideration. In our opinion, the small sample size may have limited statistical power to detect more nuanced differences between these groups. Nonetheless, the specific subscale findings suggest that the ASI may still be useful in capturing early psychosis‐related processes in heterogeneous clinical populations.

Finally, no statistically significant differences in ASI scores between ASD and APS were observed. This result reinforces the strong correlation between ASD and psychosis and highlights the marked psychotic vulnerability of this patient group. It also aligns with recent findings suggesting that autistic traits and psychotic‐like experiences may share a common neurodevelopmental substrate (Lai et al. 2019) and underscores the importance of further investigating the psychotic dimension within the ASD population (Ribolsi et al. 2023).

Even if alterations in the SN have been identified in individuals with ASD (Jao Keehn et al. 2021; Chen et al. 2017), suggesting potential neurobiological underpinnings of AS processes in this population, the ASI score is highly correlated to quantitative measures of schizotypy and positive schizotypal traits (e.g., odd beliefs, referential thinking, suspiciousness and unusual perceptual experiences) (Merola et al. 2023; Chun et al. 2019). Moreover, previous studies have reported significant correlations between autistic symptoms and schizotypal traits (Barneveld et al. 2011). These findings highlight the need for targeted research to refine diagnostic approaches and develop tailored interventions that address psychotic‐like experiences in ASD and neurodevelopmental disorders (Ribolsi, Fiori Nastro, et al. 2022; Ribolsi, Albergo, et al. 2022; Ribolsi et al. 2024).

In line with this, the longitudinal observation of the APS group further supports the clinical utility of the ASI in multi‐stage screening strategies (Fiori Nastro, Clemente, et al. 2024; Raballo et al. 2019) and keeps us reflecting on the close relationship between ASD and individuals with full‐blown psychosis.

Finally, given the relationship between AS and depressive symptoms (Azzali et al. 2022; Fiori Nastro, Pelle, et al. 2023; Lisi et al. 2021), our findings may also be interpreted in the context of the high prevalence of comorbid depressive symptoms in both CHR‐P and ASD populations. In this regard, Poletti et al. (Poletti et al. 2019) hypothesised that AS could represent a multi‐potential ‘*pathoplastic*’ factor contributing to the development of various mental disorders beyond psychosis (e.g., anxiety disorders and major depression). Additionally, AS may function as a state indicator of psychotic‐like distress, in addition to its historically postulated role as a trait indicator of vulnerability to psychosis, closely linked to schizotypy (Poletti et al. 2022).

### 4.1 Strengths and Limitations

The study has several limitations and strengths that should be considered. First, the small sample size may limit the generalizability and replicability of the findings in larger populations. Second, the cross‐sectional design precludes conclusions about how different AS profiles influence the clinical course and functional outcomes over time. Third, as this was an exploratory study with no a priori hypotheses regarding effect sizes, a formal power analysis was not conducted. Additionally, reliance on a self‐report measure of AS is a limitation, underscoring the need for further studies incorporating neurocognitive (task‐based) assessments.

A key strength of this study is the exclusion of individuals receiving antipsychotic treatment, which reduces potential confounding effects associated with pharmacological interventions.

Future research employing larger sample sizes and longitudinal designs is needed to address the limitations. Moreover, examining transition rates to full psychosis in individuals with ASD and comparing them to those in the APS group may yield further insights into the psychopathological overlap between ASD and psychosis‐spectrum conditions (Ribolsi, Fiori Nastro, et al. 2022; Riccioni et al. 2022).

## Conflicts of Interest

The authors declare no conflicts of interest.

## Supporting information


**Tables S1:** Non‐parametric ANCOVA for the ASI scores in the ASD vs. APS vs. help seeker groups. Age and years of education were entered as covariates. Significant *p*‐values are in bold.
**Tables S2:** Non‐parametric ANCOVA for the ASI scores in the ASD vs. Converter vs. Non‐Converter vs. help seeker groups. Age and years of education were entered as covariates. Significant *p*‐values are in bold.

## Data Availability

Research data are not shared.
