# Supplementary material for: Investigating Aberrant Salience in Autism Spectrum Disorder and Psychosis Risk: A Cross‐Group Analysis
Source: Early Interv Psychiatry. 2025 Oct 6;19(10):e70099. doi: 10.1111/eip.70099 (PMC12500355; doi:10.1111/eip.70099)
Supplement: Supplementary file 1 — Tables S1: Non‐parametric ANCOVA for the ASI scores in the ASD vs. APS vs. help seeker groups. Age and years of education were entered as covariates. Significant p‐values are in bold. Tables S2: Non‐parametric ANCOVA for the ASI scores in the ASD vs. Converter vs. Non‐Converter vs. help seeker groups. Age and years of education were entered as covariates. Significant p‐values are in bold. [file EIP-19-0-s001.docx]

**Supplementary Material**

**Tables S1:** Non-parametric ANCOVA for the ASI scores in the ASD vs. APS vs. help seeker groups. Age and years of education were entered as covariates. Significant p-values are in bold.

ASI Total

|  | **Df** | **F value** | **p** |
| --- | --- | --- | --- |
| Age | 1 | 0.024 | 0.8765 |
| Education | 1 | 3.819 | 0.0536 |
| Group (ASD-APS-help-seekers) | 2 | 3.428 | **0.0366** |

Feelings of Increased Significance

|  | **Df** | **F value** | **p** |
| --- | --- | --- | --- |
| Age | 1 | 0.133 | 0.717 |
| Education | 1 | 0.509 | 0.477 |
| Group (ASD-APS-help-seekers) | 2 | 1.195 | 0.307 |

Sense Sharpening

|  | **Df** | **F value** | **p** |
| --- | --- | --- | --- |
| Age | 1 | 0.021 | 0.885161 |
| Education | 1 | 15.401 | **0.000165** |
| Group (ASD-APS-help-seekers) | 2 | 9.064 | **0.000252** |

Impending Understanding

|  | **Df** | **F value** | **p** |
| --- | --- | --- | --- |
| Age | 1 | 0.003 | 0.9581 |
| Education | 1 | 4.619 | **0.0342** |
| Group (ASD-APS-help-seekers) | 2 | 3.708 | **0.0282** |

Heightened Emotionality

|  | **Df** | **F value** | **p** |
| --- | --- | --- | --- |
| Age | 1 | 0.161 | 0.689 |
| Education | 1 | 1.977 | 0.163 |
| Group (ASD-APS-help-seekers) | 2 | 1.792 | 0.172 |

Heightened Cognition

|  | **Df** | **F value** | **p** |
| --- | --- | --- | --- |
| Age | 1 | 0.099 | 0.7542 |
| Education | 1 | 6.557 | **0.0120** |
| Group (ASD-APS-help-seeker) | 2 | 4.362 | **0.0154** |

Reported values include degrees of freedom (Df), F statistics, and associated p-values.

**Tables S2:** Non-parametric ANCOVA for the ASI scores in the ASD vs. Converter vs Non-Converter vs. help seeker groups. Age and years of education were entered as covariates. Significant p-values are in bold.

ASI Total

|  | **Df** | **F value** | **p** |
| --- | --- | --- | --- |
| Age | 1 | 0.027 | 0.87089 |
| Education | 1 | 4.179 | **0.04375** |
| Group (ASD-Converters-Non Converters-help-seekers) | 3 | 5.785 | **0.00114** |

Feelings of Increased Significance

|  | **Df** | **F value** | **p** |
| --- | --- | --- | --- |
| Age | 1 | 0.143 | 0.7060 |
| Education | 1 | 0.549 | 0.4604 |
| Group (ASD-Converters-Non Converters-help-seekers) | 3 | 3.697 | **0.0145** |

Sense Sharpening

|  | **Df** | **F value** | **p** |
| --- | --- | --- | --- |
| Age | 1 | 0.021 | 0.883837 |
| Education | 1 | 15.762 | **0.000141** |
| Group (ASD-Converters-Non Converters-help-seekers) | 3 | 7.251 | **0.000201** |

Impending Understanding

|  | **Df** | **F value** | **p** |
| --- | --- | --- | --- |
| Age | 1 | 0.003 | 0.95666 |
| Education | 1 | 4.945 | **0.02859** |
| Group (ASD-Converters-Non Converters-help-seekers) | 3 | 5.187 | **0.00234** |

Heightened Emotionality

|  | **Df** | **F value** | **p** |
| --- | --- | --- | --- |
| Age | 1 | 0.173 | 0.6788 |
| Education | 1 | 2.117 | 0.1490 |
| Group (ASD-Converters-Non Converters-help-seekers) | 3 | 3.835 | **0.0123** |

Heightened Cognition

|  | **Df** | **F value** | **p** |
| --- | --- | --- | --- |
| Age | 1 | 0.109 | 0.74212 |
| Education | 1 | 7.244 | **0.00844** |
| Group (ASD-Converters-Non Converters-help-seekers) | 3 | 6.827 | **0.00033** |

Reported values include degrees of freedom (Df), F statistics, and associated p-values.
